# Apoplexy Caused (as Was Supposed) by Gymnastic Exercises, with Criticisms

**Published:** 1829-01-01

**Authors:** 


### XXXIII.

### Apoplexy caused (as was supposed) by Gymnastic Exercises.

In the first number of Dr. Farre's half-yearly Journal, there is reported a case of the above description. A young man, aged 20 years, of muscular frame, short neck, and middle size, had habituated himself to considerable gymnastic exercises, and never required the assistance of a medical man. On rising from his bed, on the 19th of October, 1826, he fell down insensible, and expired in the course of an hour. The head was examined the same day. On separating the skull-cap from the dura mater, three or four ounces of blood escaped. The vessels of the pia mater were turgid—the convolutions of the brain had a flattened appearance. In the right hemisphere of the brain there was a clot of blood, and also an effusion of fluid blood to the amount of five or six ounces. The lateral ventricles were also found filled with blood. The texture of the brain was, of course, completely broken up in the part where the blood was extravasated.

We wonder that so grave and accurate a reasoner as Dr. Farre, should set down the above extravasation of blood, as "consequent on habitual gymnastic exercises." This is an exquisite specimen of the "post hoc, ergo propter hoc" ratiocination. The narrator might just as well infer, that the death of every old Greenwich pensioner is the consequence of a sea-life—the said sea-life having, *in every instance*, preceded the death of the individual! Or, going to Chelsea, and examining the necrological records there, he might deplore the dreadful effects which result from carrying a musket—all the men who die at Chelsea having "habitually" borne arms.

If the reader will turn (in the same Journal) to the case which immediately precedes the one we have been commenting on, he will find an instance of sanguineous effusion in the brain of another young man—"tall, muscular, and *accustomed to drink spirits*." He died suddenly, and besides six ounces of water and some blood in the ventricles, there were four ounces of coagulated blood at the base of the brain, which blood had escaped "from a small aneurism of the left vertebral artery." Both vertebral arteries were in a diseased state, being, in some places, cartilaginous, though the individual was only 24 years of age. The communicator of this case has not, though he *ought* to have drawn the conclusion, that the aneurism of the vertebral artery was "consequent on dram-drinking." We are ashamed to see such hasty and unsupported inferences drawn in medicine.

---

